# Quantitative Study on the Impact of Energy Consumption Based Dynamic Selfishness in MANETs †

**DOI:** 10.3390/s21030716

**Published:** 2021-01-21

**Authors:** Axida Shan, Xiumei Fan, Celimuge Wu, Xinghui Zhang, Shujia Fan

**Affiliations:** 1 Department of Automation and Information Engineering, Xi’an University of Technology, Xi’an 710048, China; axida@bttc.edu.cn (A.S.); 1180311021@stu.xaut.edu.cn (X.Z.); 2 School of Information Science and Technology, Baotou Teachers’ College, Baotou 014030, China; 3 Graduate School of Informatics and Engineering, The University of Electro-Communications, Tokyo 182-8585, Japan; celimuge@uec.ac.jp; 4 College of Electronic & Information Engineering, Ankang University, Ankang 725000, China; 5 Department of Computer Science and Technology, Tsinghua University, Beijing 100084, China; kikifan@bu.edu

**Keywords:** selfishness, MANET, impact, energy consumption, OMNeT++

## Abstract

Cooperative communication and resource limitation are two main characteristics of mobile ad hoc networks (MANETs). On one hand, communication among the nodes in MANETs highly depends on the cooperation among nodes because of the limited transmission range of the nodes, and multi-hop communications are needed in most cases. On the other hand, every node in MANETs has stringent resource constraints on computations, communications, memory, and energy. These two characteristics lead to the existence of selfish nodes in MANETs, which affects the network performance in various aspects. In this paper, we quantitatively investigate the impacts of node selfishness caused by energy depletion in MANETs in terms of packet loss rate, round-trip delay, and throughput. We conducted extensive measurements on a proper simulation platform incorporating an OMNeT++ and INET Framework. Our experimental results quantitatively indicate the impact of node selfishness on the network performance in MANETs. The results also imply that it is important to evaluate the impact of node selfishness by jointly considering selfish nodes’ mobility models, densities, proportions, and combinations.

## 1. Introduction

With the pervasiveness of Internet of Things (IoT) and the rapid development of communication technologies, mobile ad hoc networks (MANETs) have become more and more important in IoT-related areas, especially in smart IoT. MANET is a wireless network that consists of a number of autonomous, self-organized, limited energy capacity, and mobile nodes, which communicate with each other over wireless communication links in an ad hoc manner without the assistance of any centralized authority [1]. MANET has a wide usage in industrial applications and other scenarios that requires the immediate establishment communication with dynamic survival networks. Every node in a MANET functions as both host, which acts as a normal terminal device, and router, which forwards packets to assist routing operations. These nodes can be any personal device such as laptops, mobile phones, or tablets that are battery-driven in general [2].

Due to lack of centralized authorities and limited transmission range of wireless communication links, most communications among the nodes in MANETs highly depend on multi-hop routing mechanisms. As a result, forwarding tasks become critically important during the communication process [3]. However, the resource-constrained mobile nodes in MANETs are unwilling to forward data packets for others’ interests in order to conserve their own resources.

In general, the nodes in the network are divided into three categories: normal, malicious, and selfish nodes. Normal nodes always operate network functionalities in a cooperative way. Malicious nodes intentionally damage others by causing network outages, while saving resources is not a priority. Selfish nodes take advantage of the network to send/receive packets with their own interests, but they refuse to forward packets for others because of the limited computation and communication resources. They do not have direct intentions to damage other nodes or the network. However, the selfish nodes in MANETs have great impacts on the overall network performance.

**Figure 1:**
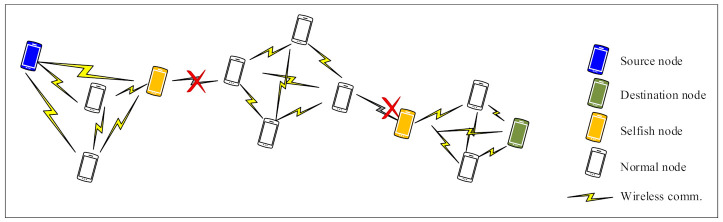
An example of network partitioning caused by the presence of selfish nodes in a mobile ad hoc network (MANET).

This paper mainly focuses on the evaluation of the impact of node selfishness on the network performance in MANETs. Selfishness is a most common passive denial of service (DoS) attack that lowers the network performance [4]. As clearly depicted in Figure 1, the presence of selfish nodes causes negative implications on the network (e.g., network partitioning problems).

In most literature, the impacts of selfish nodes on routing protocols, detection, or incentive mechanisms are the main topics [5,6,7]. However, we are interested in investigating the selfish node impacts on the overall network performance because the quantification of the investigation could provide reliable information to detection and incentive mechanisms. In this study, we hypothesize that mobility, density, proportion, and combination of selfish nodes are related to the overall network performance, and we utilize computer simulation based experiments to test this hypothesis. All the related issues are discussed in detail in the following sections from various aspects.

This paper is partially based on our previous conference paper [3] where the impacts of selfish nodes in MANETs were studied. However, the selfish nodes discussed in the previous work were all static selfish nodes, which means the selfishness is unchanged during the whole process. In fact, static selfishness is not practical in the real world. In order to more realistically investigate node selfishness in MANETs, energy consumption based dynamic selfishness is discussed in this paper. The major contributions of our work are summarized as follows.

- To the best of our knowledge, this is the first work in which the dynamic selfishness is quantitatively studied by considering mobility, density, proportion, and combination of selfish nodes in MANETs and evaluated from various aspects including average packet loss rate, average round-trip delay, and throughput.
- We defined energy consumption based dynamic selfishness, which is less harmful but more realistic in MANETs, and its impacts on the performance of network are evaluated from various aspects with comparison to that of static selfish nodes as baselines.
- In this work, a static/dynamic selfishness switching functionality is implemented in the routing table module at Network Layer in INET Framework [8], and it is irrelevant to any specific routing protocol implementation. Therefore, there is no need to customize routing protocols to test the network performance with static/dynamic selfish nodes.
- Exhaustive computer simulations are conducted on a proper platform to evaluate the impacts of selfish nodes with various randomization in MANETs.

The remainder of the paper is organized as follows: In Section 2, we discuss some recent work related to node selfishness in wireless ad hoc networks. The energy consumption based dynamic selfish nodes are defined in Section 3. Evaluation algorithms and evaluation metrics are introduced Section 4. Simulation setup including the platform and parameters used in this work is given Section 5. Section 6 provides the results of the simulations and corresponding explanations. The results obtained from the simulations are discussed in Section 7. Finally, Section 8 concludes our work and points out our future research directions.

## 2. Related Work

In recent years, a number of aspects related to node selfishness in wireless networks have been extensively investigated in various aspects. The research works in the literature generally fall into following three categories: selfish nodes detection, incentive mechanisms for selfish nodes, and impact analysis of selfish nodes.

### 2.1. Selfish Node Detection

Selfish node detection techniques in wireless ad hoc networks have attracted many researchers recently. Aifa et al. [6] reviewed several different selfish nodes detecting methods including Watchdog and Pathrater approaches in MANETs. Although the Watchdog approach can identify the misbehavior node at the forwarding level, it is not able to detect the misbehavior node in collision situations. The Pathrater approach eliminates the route containing misbehavior nodes from the routing protocol. Vij et al. [9] proposed a detection scheme for selfish nodes in MANETs based on game theories. The proposed protocol uses the battery as a limited resource in routing and identifies selfish nodes with their battery consumption. However, the energy generation and consumption are not adequately discussed. Lupia et al. [10] proposed a selfish node monitoring and detecting system with reduction in energy consumption and without lowering detection performance. RoselinMary et al. [11] proposed an Attacked Packet Detection Algorithm (APDA) in vehicular ad hoc networks (VANETs) that uses node position, velocity, frequency, and the number of packets broadcast per second, attached to road side unit (RSU), to detect DoS attacks before verification time. Singh et al. [12] further developed Enhanced Attacked Packet Detection Algorithm (EAPDA) in VANETs, upgraded the algorithm by the improvement of throughput. Kim et al. [13] proposed a collaborative security attack detection mechanism based on multi-class support vector machine (SVM) in a software-defined vehicular cloud (SDVC) environment. However, the mechanism assumed that all vehicles in the network have sufficient resources for analyzing incoming flow data. Alrehan et al. [14] concluded machine learning based solutions to detect distributed denial of service (DDoS) attacks on VANET systems. Furthermore, Ilavendhan et al. [15] analyzed various state-of-the-art approaches for DoS attack detection in VANETs. However, in the abovementioned literature, the impact of selfish nodes, the focus of this paper, is not discussed adequately in neither MANETs nor VANETs.

### 2.2. Incentive Mechanisms for Selfish Nodes

To reduce the harmful effects on the network caused by the presence of selfish nodes, encouraging nodes to cooperate is critical to ensure the network functions properly. In general, incentive mechanisms can be classified into the following three main kinds: reputation-based, credit-based, and barter-based systems.

The main idea of a reputation-based incentive mechanism is that more cooperative nodes get higher reputation scores [16]. The most challenging issue of the reputation-based incentive mechanism is to accurately measure the reputation scores to each node in the network. Wu et al. [17] proposed a social norm based incentive mechanism for network coding (NC) in MANETs. A reputation system with punishment and reward is considered in the social norm. Li et al. [18] introduced a hierarchical account-aided reputation management system that integrates resource and price systems to stimulate the node cooperation in large-scale MANETs. Lai et al. [19] proposed a secure incentive scheme in highway VANETs scenarios. The scheme utilizes “virtual checks” to ensure the security and fairness of the cooperation. The authors also developed a reputation system to stimulate cooperative nodes and penalize malicious nodes. Dias et al. [20] proposed a hybrid incentive system taking advantage of both reputation mechanisms and monitoring modules in Vehicular Delay-Tolerant Networks (VDTNs). The system encourage selfish nodes to share their resources rather than excluding them from the network. Wang et al. [21] investigated a blockchain-based incentive content delivery in autonomous vehicular social networks. The reputation assessment models in the paper is based on both social features and user behaviors. In sum, reputation-based incentive mechanisms highly rely on historical information about the node behaviors, which results in the downfall of this type of incentive mechanism.

The credit-based incentive mechanism exploits some rewarding mechanisms to nodes for indicating cooperativeness [22]. Buttyán et al. [23] introduced the Nuglet technique. The authors proposed a credit-based incentive protocol that requires the node to forward each packet to its security module in MANETs. The security module, nuglet, is a counter for each node. The nuglet increases (decreases) when the node sends its own (other’s) packets. Meeran et al. [24] proposed an enhanced selfish node detection system based on a watchdog mechanism in MANETs. The system revives selfish nodes in the network, instead of isolating them. A virtual payment (credit) is defined in the system, and forwarding nodes will get credit while selfish nodes will get debited. If a node does not have enough credit, it cannot act as a source node to send packets.

The barter-based strategy is also known as Tit-for-Tat (TFT) to punish uncooperative nodes. In a TFT strategy, a node takes cooperative or selfish action according to the action from the previous node. Each node in the network represents a player in game theories. Hence, the barter-based strategy is also considered as a game theory approach. Wu et al. [25] proposed a reward allocation mechanism based on the integration of game theory and reinforcement learning algorithms to maximize the whole network performance. Li et al. [26] integrated a reputation system and a price-based system for selfish node detection and incentives in MANETs with a game theory perspective. Khan et al. [27] introduced an evolutionary game theory based intelligent packet forwarding approach that stimulates the node cooperation in MANETs. Yang et al. [28] proposed a Stackelberg game based optimal pricing strategy to model data offloading in VANETs scenarios. AI-Terri et al. [29] proposed two TFT-based strategies, namely Group Reputation and Cooperative Detection strategies, to enforce MAC layer cooperation in VANETs. The proposals address the greediness problem and achieve better misbehavior detection performance.

The main concern of the abovementioned incentive schemes is to stimulate or punish misbehaving nodes in the network. However, the impact of selfish nodes on overall network performance was not quantitatively analyzed in the literature.

### 2.3. Impact Analysis of Selfish Nodes

Although a wide range of investigations have been done on node selfishness in various networks, very little research has been devoted to analyze the impact of node selfishness on network performance in MANETs.

In earlier work [30], Kyasanur et al. introduced that some hosts may misbehave by failing to adhere to the network protocols at the MAC layer. The authors also presented detection and penalty schemes to handle the misbehavior successfully. Later, Lei et al. [31] measured the performance of two popular routing protocols (AODV and DSR) under MAC layer selfish attacks in MANETs. The authors suggest that cross-layer cooperation between MAC layer and network layer could be one feasible solution to mitigate the MAC attack effects. Xu et al. [32] analyzed the effect of node selfishness in MANETs. The authors mainly considered two kinds of selfish nodes, namely type-1 and type-2. The type-1 model is the model in which the selfish nodes do not forward packets, while in the type-2 model, the selfish nodes do not take part in the routing operations. According to this work, it is obvious that the node selfishness is more harmful to network performance in the type-2 model than in the type-1 model. Kampitaki et al. [33] investigated the functions of DSR protocol, and they defined several kinds of selfish nodes to examine their impacts on the network performance. Recently, Loudari et al. [34] studied the effects of selfishness on the energy consumption in opportunistic networks (OppNet). This work mainly investigated different impacts of selfishness on energy consumption under OppNet routing protocols. The authors acknowledged that the remaining energy in the node can play a crucial role in its willingness to cooperate in the network. However, their work lacked quantitative analysis of node selfishness in terms of presence, mobility, and density. Quantitative analysis of node selfishness based on energy consumption in MANETs is still an open issue, and more work is still required.

## 3. Energy Consumption Based Selfish Nodes

### 3.1. Selfish Nodes

Selfishness is a normal behavior that is present in all aspects of life, and MANET is not an exception. The term “selfish node” appears in the work of Marti et al. [35]. In this paper, a selfish node is the node that takes advantage of the network by sending and receiving data for its own interests. However, it behaves selfishly not to forward data, even the routing control packets, but for other nodes in order to reduce the power consumption.

There are two types of selfish nodes considered in this paper: static selfish nodes and dynamic selfish nodes. Static selfish nodes are those where their selfishness is static (unchanging) during the whole network procedure. Dynamic selfish nodes are nodes where their selfishness dynamically changes depending on some conditions.

### 3.2. Energy Consumption

In MANETs, the main energy consumer in the mobile nodes is the transceiver. In this work, energy consumption is based on power consumption values for various transceiver modes, states, and the time the transceiver spends in these states. Transceivers consume a small amount of power when they are idle in receiving mode. They consume more when they are receiving a transmission, and even more when they are transmitting.

**Figure 2:**
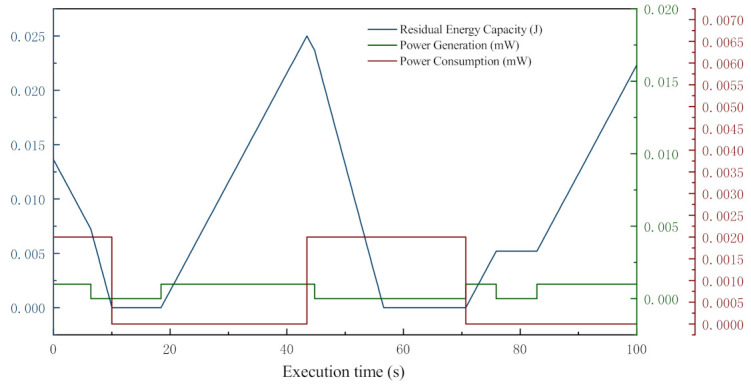
An example of single node energy variation.

In order to clearly illustrate the energy variation of a node in MANETs, the related data of a normal node are collected from a simple test and plotted in Figure 2.

In this test, the nominal energy capacity of the node is 0.025 J, initial residual energy is a random value between 0 J and 0.025 J, and the test lasts 100 s. The graph clearly visualizes the changes in energy generation, consumption, and storage. In the figure, the energy generation, consumption, and storage are indicated by the green, red, and blue solid lines, respectively. The node starts from a given charge level, and its energy level constantly decreases from there. It eventually reaches the shutdown capacity (0 J), and when that happens, the node shuts down. Then it starts to charge, and when the charge level reaches the start capacity, the node turns back on. When the transceiver in the node consumes energy by receiving/transmitting operations, the energy storage decreases fast. However, when the node is transmitting and the generator is charging, the energy level does not decrease as fast. In contrast, when the node is in the idle state and the generator is charging, the storage level increases linearly.

In sum, it is evident that residual energy of a node can represent the limited resources in a dynamic way, which make nodes selfish in MANETs. That is also the reason we define energy consumption based dynamic selfishness in this work.

### 3.3. Energy Consumption Based Dynamic Selfish Nodes

In order to acquire more realistic evaluation results, energy consumption based dynamic selfishness is defined in this paper, and its impact on the network performance is evaluated by comparison with static one as a baseline.

A node functions altruistically if its remaining energy is always higher than a threshold during the entire procedure. In contrast, a node functions as a static selfish node if its remaining energy is always lower than the threshold. However, an energy consumption based dynamic selfish node alters its states between selfish and altruistic depending on its remaining energy. Most nodes in MANETs are battery-powered. The remaining energy is the precious resource of the nodes in MANETs, which has the highest priority to preserve. Therefore, it is reasonable to assume that nodes behave selfishly only when its remaining energy is lower than a certain value. In other words, the remaining energy of a node represents the integration of all resources it possesses. These definitions make the evaluation more realistic and its results more reliable.

A switching functionality is implemented at Network Layer in our experiments to simulate the energy consumption based dynamic selfishness. Specifically speaking, whenever the routing protocol checks the routing table to forward a packet, the switching functionality is triggered to determine whether to forward it by calculating the remaining energy capacity. The packet is forwarded if the remaining energy is greater than the selfish threshold. Otherwise, the packet is simply dropped by the routing protocol.

In this work, the node states are determined by the residual energy capacity (REC). A node shuts down when its REC is lower than *D*th (power off threshold), restarts it when its energy storage charge reaches *U*th (power on threshold), and becomes selfish when its remaining charge is lower than *S*th (selfish threshold) as shown in Equation (1).

(1) $$\mathrm{Node}\;\mathrm{State}\;:=\left\{\begin{array}{cc} up, & \mathrm{if}\;REC\geq U_{th}\\ selfish, & \mathrm{if}\;D_{th}<REC\leq S_{th}\\ down, & \mathrm{if}\;REC\leq D_{th} \end{array}\right.$$

## 4. Evaluation Methods

### 4.1. Evaluation Algorithm

The impact of node selfishness is examined by running a simple User Data Protocol (UDP) application on the Transport Layer. The application is regarded as UDP network traffic. The sender node generates ping requests to the destination node and calculates the packet loss and round-trip times of the replies. It works exactly like the ‘ping’ command in most operating systems. Every ping request is sent out with a sequence number, and replies are expected to arrive in the same order. Whenever there is a jump in the responses’ sequence number, then the missing pings are counted as lost. Then if it still arrives later, it will be counted as out-of-sequence arrival, and at the same time the number of losses is decremented [8].

**Figure 3:**
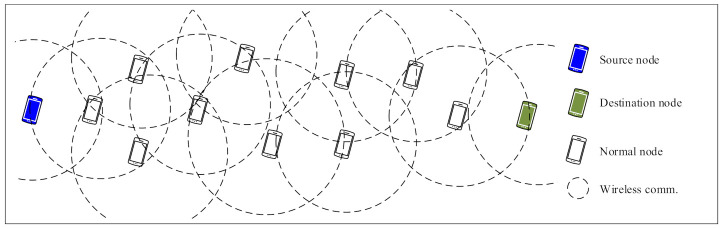
Evaluation algorithm scenario.

As illustrated in Figure 3, the sender (source) node and receiver (destination) node are fixed at left and right side of the constraint area, respectively. Both the sender and receiver are out of the transmission range of each other, which means they need at least one intermediate node to communicate with each other. All other nodes are randomly distributed between the sender and receiver node and arbitrarily move in the constraint area. It means that all nodes except for the sender and receiver are intermediate nodes bearing forwarding tasks.

In this evaluation algorithm, selfish behaviors of nodes are more noticeable because the only communication task the nodes perform is data forwarding. In order to estimate the overall communication performance effected by selfish nodes, more general evaluation algorithms in which more senders and receivers are deployed are planed in our future work.

### 4.2. Evaluation Metrics

Data forwarding is the fundamental network function in MANETs. In order to estimate the impact of node selfishness on the network performance based on packet forwarding efficiency, three metrics are used, namely average packet loss rate, average round-trip delay, and average throughput.

(1) Average Packet Loss Rate (APLR): This metric is calculated as the ratio of the number of lost data packets to the number of all packets sent by the source nodes.

(2) $$APLR=\frac{P_{lost}}{P_{sent}}*100\%$$

In Equation (2), $P_{sent}$ and $P_{lost}$ represent the number of packets sent by the source node and the number of packets that did not reply, respectively.

(2) Average Round-Trip Delay (ARTD): This metric is calculated as the average sum of the round-trip delay of each data packet that the source node sent.

(3) $$ARTD=\frac{\sum_{i=1}^{P_{sent}-P_{lost}}\left[T_{r}\left(i\right)-T_{s}\left(i\right)\right]}{P_{sent}-P_{lost}}$$

In Equation (3), $T_{r}\left(x\right)$ represents the time when the *i*th corresponding reply packet is received by the sender, $T_{s}\left(x\right)$ represents the time when the *i*th data packet is sent out of sender *x*.

(3) Average Throughput (AT): This metric is calculated as the sum of successfully received packet sizes divided by the simulation time.

(4) $$AT=\frac{\sum_{i=1}^{P_{sent}-P_{lost}}P_{size}}{T_{b}-T_{e}}$$

In Equation (4), $P_{size}$ represents the packet size, and $T_{b}$ and $T_{e}$ are the simulation start time and the end time, respectively.

## 5. Simulation Setup

### 5.1. Simulation Platform

In order to quantitatively evaluate the impact of node selfishness caused by energy consumption in MANETs, we designed the simulation procedure using a proper platform and frameworks.

For the purposes of our investigation, the integration of OMNeT++ (v5.5.1) [36] and INET Framework (v4.1.1) [8] is used as the simulation platform in this paper. OMNeT++ is an open source computer simulation platform written in C++ that is suitable for wireless and discrete network event simulations. INET Framework is an open source framework for the OMNeT++ platform. It provides adequate implementations of communication network protocols.

### 5.2. Simulation Parameters

The general simulation parameters are shown in Table 1. Each simulation runs for 500 s. The simulation area is 300 m × 1000 m, and the node movements follow a random waypoint mobility (RWP) model [37], which is the most common used one in related articles. At the initial stage of simulations, all nodes are randomly distributed in the constraint area.

In this paper, the IdealWirelessNic module from INET Framework is used instead of a specific MAC layer protocol implementation. This module is a highly abstracted wireless network interface card that consists of a unit disk radio and trivial medium access control protocol. We are not interested in lower-layer communication effects in this work; instead our main objective is the communication impact on the network layer. So the IdealWirelessNic module is the best choice for our simulation because of its simplicity and encapsulations.

The energy-related parameters are listed in Table 2. There are mainly three energy management modules in INET Framework, namely Energy Storage, Energy Generator, and Energy Consumer. Energy Storage is similar to a real battery and uses charge and current. The Energy Generator module alternates between active and sleep states. It starts in the active state, and while there, it generates a given amount of power. In the sleep state, it generates no power. Energy Consumer is based on power consumption values for various transceiver mode and states, and the time it stays in these states is described in Section 3.2.

Note the following in our simulations:

- Each error bar shows the 95% confidence interval of the corresponding data.
- The number of nodes does not include the sender and receiver; it includes intermediate nodes only.
- The receiver is fixed far from the sender, and the multi-hop is the only way to communicate with each other.
- Dynamic selfish nodes mean the nodes behave selfishly only if the residual energy level is lower than the selfish threshold.
- Altruistic nodes mean the nodes behave cooperatively during whole network procedure.
- In order to intuitively express selfish thresholds, the ratio (%) of residual energy capacity to the nominal energy capacity is used in our simulation instead of $S_{th}$ defined in Joules.

## 6. Experimental Results

### 6.1. Impact of Selfishness on Energy Consumption

Altruistic (normal) nodes in MANETs behave cooperatively without considering the remaining energy in the storage, while selfish nodes unfold their selfishness whenever it lacks energy during network operations. In order to investigate how much energy could saved by selfish behaviors, the following comparison experiments are conducted.

**Figure 4:**
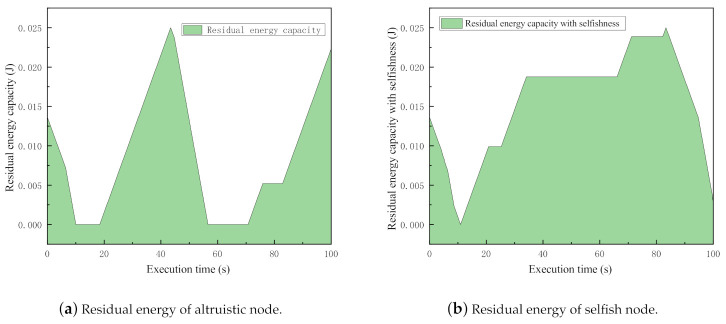
Comparison of residual energy between altruistic and selfish nodes.

In the experiments, there are sender, receiver, and 10 intermediate nodes deployed. The simulation time is set as 100 s. Two sets of simulations are executed. In the first set, all intermediate nodes are set as normal nodes, while in the second one, the nodes are set as selfish ones. With considering a fair comparison, we compare the same nodes from each simulation because they have the same settings except for the selfishness. The residual energy levels during the simulation procedure are plotted in Figure 4.

Obviously, when nodes are selfish, the accumulation of residual energy is much larger than altruistic energy. Quantitatively speaking, the accumulated residual energies of the selfish and altruistic nodes are 3.474881833 (J) and 2.128937979 (J), respectively. The node saved about 1.345943854 (J) energy in 100 s by behaving selfishly. This experiment quantitatively explored the motivation of node selfishness.

### 6.2. Impact of Mobility Models

One of the key characteristics of wireless ad hoc networks is mobility. Mobility does indeed affect the performance of the network. Although the investigation of various mobility models is out of the scope of this paper, it is still worth to carry out some experiments to clarify the impact of mobility in MANETs. Note that the network communication performance is negatively influenced by not only the node selfishness but also the mobility models. In order to assess how the mobility model affects the communication performance of the network, we conducted the simulation with and without mobility.

**Figure 5:**
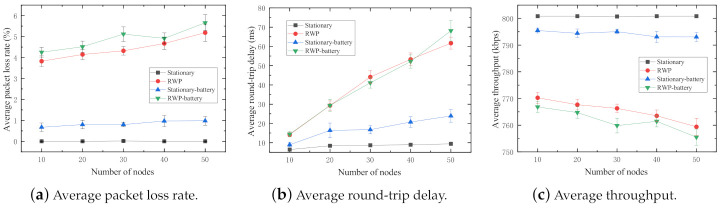
Performance impact of random waypoint mobility.

In the experiment, four sets of simulations are conducted. Note that all nodes in this experiment are normal nodes. (1) Stationary: All nodes in the network are randomly scattered and immobile at the initial positions. (2) RWP: All nodes in the network follow the random waypoint mobility model. (3) *Stationary-battery*: All nodes in the network are Stationary with considering energy consumption. (4) *RWP-battery*: All nodes in the network are RWP with considering energy consumption. The number of nodes varies from 10 to 50 with step size of 10. The results of the experiment are shown in Figure 5.

It is clear in Figure 5a that the packet loss rate is significantly increased by the node mobility. It reflects the fact that in a MANET, link breakages more likely occur because of dynamic topologies caused by the node mobility. In contrast, the packet loss rates in the stationary model keep almost 0% during whole simulation process. It means that there is at least one routing path available in most cases of random node distributions, which makes sure all packets sent by the sender are successfully received by the receiver. In addition, *Stationary-battery* and *RWP-battery* lost more packets than Stationary and RWP because some battery-powered nodes could run out of battery and crash during the simulation, which causes unexpected link breakages in the network.

The average round-trip delay of the nodes with random waypoint (RWP, *RWP-battery*) mobility drastically increases with the increase in number of nodes in Figure 5b. This is because the greater the number of mobile nodes involved the network operations, the longer the routing path needed to reach the destination. In other words, the routing protocol spends more time on route discovery and route maintenance operations because of the dynamic topology. The increments in hop counts result in long round-trip delays. Additionally, the sum of routing procedure time spent on every node in the route linearly increases with the number of nodes in the route. In the case of stationary nodes (Stationary, *Stationary-battery*), with the increasing total number of nodes in the network, the number of neighbors (one-hop distant node) of every node increases, which changes the nearest node in the communication range and slightly affects the round-trip delays. Last but not least, the initial position of nodes is a non-negligible factor to influence the average round-trip delay, especially in stationary networks. These are the reasons of the slight increments in average round-trip delay when the nodes in the network are stationary. However, the average round-trip delay of the stationary nodes is significantly less than that of the nodes with random waypoint mobility. This is because routing table update operations seldom occur in the stationary mobility network, which further saved a large amount of time to discover new routes and constantly update the routing table in every node.

From Figure 5c, we can observe that the average throughput of the stationary network is much higher than that of random waypoint in both networks with and without energy consumption considered. In other words, the bandwidth utilization of the stationary network is more efficient than that of random waypoint. This primarily is due to the low packet loss rates (refer to Equation (4)).

In sum, as a key characteristic, the mobility of nodes has significant, negative impacts on the network performance of MANETs.

### 6.3. Impact of Selfish Node Densities

In general, node density is defined as the number of nodes per unit area. It is reasonable to define node density as the number of nodes in the simulation because the simulation area is fixed. In order to evaluate the impact of selfish nodes in various density networks, the following experiments are conducted.

**Figure 6:**
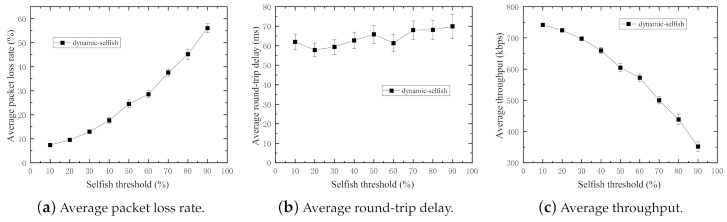
Performance impact of various selfish thresholds (50 nodes).

For the purpose to assess the impact of different selfish thresholds on the performance of networks, the evaluation algorithm is executed with different selfish thresholds varying from 10% to 90% with the step size of 10% (0% and 100% are excluded because they mean altruistic and static selfish, respectively), and the number of nodes is fixed at 50. The results of the simulation are shown in Figure 6.

In Figure 6a,c, it is obvious that the average packet loss rate increases with the increasing selfish thresholds. That means the higher threshold results in more selfish nodes in the network. The average throughput decreases with the increasing selfish thresholds because of increments in packet loss rates.

Figure 6b indicates there is little impact on average round-trip delay because average round-trip delay is mostly relevant to the packets successfully sent the destination (receiver). However, there are small fluctuations in the figure, which are because of dynamic selfishness and high mobility of the nodes. The dynamic selfish nodes in this work do not forward user data packets nor routing control packets. Therefore, the route discovery operations of the routing protocol are slightly influenced by the randomness of the selfish nodes.

Because the residual energy level of each node is time-varying, the node states alternate between selfish and altruistic dynamically. Furthermore, it can be statistically deduced that the residual energy of most nodes is at a much higher level because the increments of average packet loss rates become larger with increasing selfish thresholds. Accordingly, the remaining experiments in following subsections are carried out with the fixed selfish threshold at 30%.

**Figure 7:**
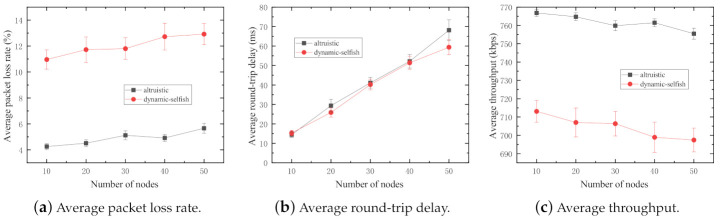
Performance impact of various node densities (selfish threshold: 30%).

Subsequently, the three evaluation metrics are compared in the MANET with various numbers of nodes, i.e., various node densities. The number of nodes varies from 10 to 50 with the step size of 10. Two sets of simulations are conducted for the purpose to compare the impact of selfish nodes to that of altruistic nodes on the network performance of the MANET. In the first set of simulations, all nodes are altruistic during the whole procedure. In the other set of simulations, all nodes are dynamic selfish (selfish threshold is 30%). The simulation results are shown in Figure 7.

The increasing node densities give rise to increments in average packet loss rates in both sets of simulations (Figure 7a). This can be explained by the node mobility, which leads to dynamic topology changes and further results in link breakages in the network. However, there are relatively higher packet loss rates in the network with dynamic selfish nodes. In this case, nodes behave selfishly to refuse to forward packets for others, whenever the residual energy level comes down to less than the selfish threshold. The selfish behaviors lead to significant increases in the packet loss rate.

The average round-trip delay in both networks with dynamic selfish and altruistic nodes increases with increasing node densities (Figure 7b). In MANETs, the more nodes involved in the network, the more complicated network topology is. Therefore, the routing protocol discovers and manages many possible routing paths in every node, which are time-consuming tasks in MANETs. However, the average round-trip delay of the network with dynamic selfish nodes is a bit smaller than that of the network with altruistic nodes. This is because selfish nodes refuse to cooperate with the routing protocol, which could reduce the number of nodes the routing protocol deals with.

The average throughput of both networks with dynamic selfish and altruistic nodes decreases with increasing node densities in the MANET (Figure 7c). The reason is that the number of packets successfully received by the receiver is reduced because of increasing packet loss rates.

In sum, the performance of the network is significantly reduced by the increased number of selfish nodes involved in the network. The increasing node densities have negative implications on the performance of MANETs.

### 6.4. Impact of Selfish Node Proportions

Selfish nodes play negative roles in MANETs as the intermediate nodes that refuse to forward packets for others’ interests. The multi-hop communication is required in MANETs whenever a node sends a packet to the destination out of its transmission range of the node. It points out that cooperation among nodes in MANETs is indispensable in most cases. In order to make clear how the proportions of selfish nodes, including static and dynamic ones, affect the network performance in MANETs, the following simulations are conducted.

**Figure 8:**
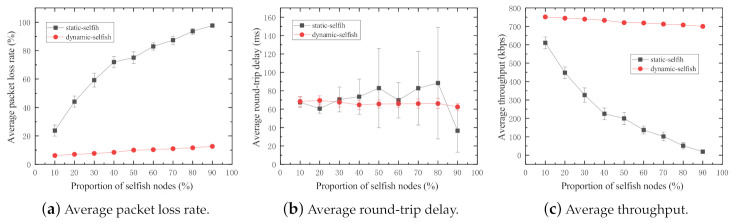
Performance impact of various proportions of selfish nodes (50 nodes).

The three evaluation metrics are investigated in the MANET with 50 intermediate nodes. In the simulations, the selfish node proportion varies from 10% to 90% with the increment of 10%. The proportions of 0% and 100% selfish nodes are excluded in the simulations for the following reasons. In the case of 0% selfish nodes, all nodes in the network altruistically cooperate with each other, which was discussed earlier in this section. In the case of 100% of selfish nodes, all nodes in the network are selfish. Especially in the network with static selfish nodes, no packet sent by the sender will reach the receiver because the receiver is out of the transmission range of the sender in our simulation setting, and there is no intermediate node to forward packets because they are static selfish. The simulation results are shown in Figure 8.

The average packet loss rate of the network increases with increasing proportions of selfish nodes in both dynamic and static selfish networks (Figure 8a). A large proportion of selfish nodes leads to a high packet loss rate in the MANET. The average packet loss rate of the network with static selfish nodes increases drastically. This significant performance reduction is caused by the fact that the absolute number of selfish nodes increases with the increasing proportion of selfish nodes in the network, which finally leads to very few intermediate nodes in the network to assist in routing and forwarding tasks. However, that of the network with dynamic selfish nodes, by contrast, does not increase obviously. The reason is that even though the proportion of dynamic selfish nodes is high, it does not mean the absolute number of selfish nodes is large. In the network with dynamic selfish nodes, the node selfishness is only determined by the dynamic residual energy level. The nodes with higher residual energy levels always act altruistically, and vice versa.

In Figure 8c, the average throughput of both networks with static and dynamic selfish nodes is depicted. The average throughput of the network with dynamic selfish nodes does not decrease too much, whereas that of the network with static selfish nodes reduces drastically. The main reason of this occurrence is the average packet loss rate.

The average round-trip delay of both networks with static and dynamic selfish nodes is depicted in Figure 8b. In the network with dynamic selfish nodes, the average round-trip delay is more stable than that in the network with static selfish nodes. In the network with static selfish nodes, as the proportion of static selfish nodes increases, the average round-trip delay becomes longer for each packet successfully received by the destination; it is more and more difficult to discover a valid route from the sender to the receiver when more and more static selfish nodes are involved in the network. However, the average round-trip delay becomes shorter when the proportion of selfish nodes is extremely high, for example 90%. From another point of view, it reflects that the number of utilizable nodes for data transmission is very small. It further results in low probabilities to establish valid routes with a small number of cooperative nodes having a high random mobility. Even if the route is established, the hop count of the route is small and the round-trip delay is short. In this simulation, the selection of selfish nodes is predetermined manually. Specifically speaking, node[0]∼node[4] are configured as selfish nodes to test 10% selfish node proportion, and node[0]∼node[9] are configured as selfish nodes to test 20% selfish node proportion, and so on. This leads to unreliable simulation results. It inspired us to investigate the impact of different combinations of selfish nodes, and the result is discussed in the following subsection.

### 6.5. Impact of Selfish Node Combinations

Theoretically speaking, if the number of topology trails is large enough, the probability of having each combination is equal. However, in practical settings, mobile users move in an organized fashion, especially in the case of VANETs, which is a type of MANET. Hence, the combination of selfish nodes is a non-negligible factor to consider in the investigation of node selfishness in MANETs. Every node in MANETs has time-varying positions, speeds, and neighbors. The different selections of selfish nodes affect the network performance variously. In fact, the evaluation of various combinations of selfish nodes synthetically indicates the joint impact of mobility, speed, and positions in MANETs.

**Figure 9:**
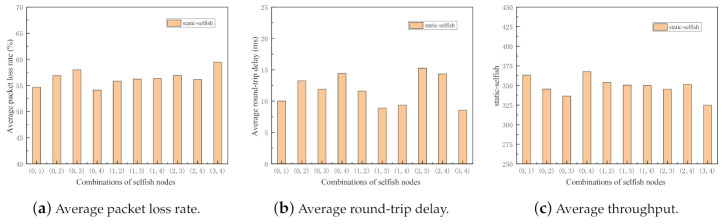
Performance impact of different combinations of static selfish nodes (5 nodes).

**Figure 10:**
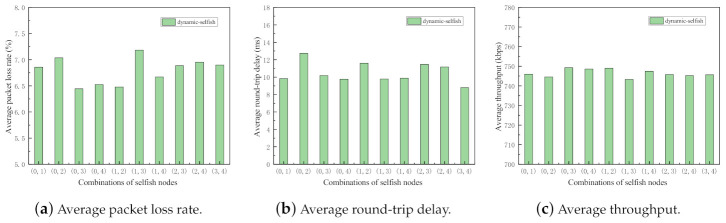
Performance impact of different combinations of dynamic selfish nodes (5 nodes).

For the purpose of the impact evaluation about the various combinations of selfish nodes, the following simulations are conducted. The evaluation metrics of the network with static and dynamic selfish nodes are investigated separately. In the simulations, five intermediate nodes are deployed with the random waypoint mobility model. In each set of simulations, there are two selfish nodes, that means 40% selfish nodes are selfish. There are 10 possible combinations, i.e., (0, 1), (0, 2), (0, 3), (0, 4), (1, 2), (1, 3), (1, 4), (2, 3), (2, 4), and (3, 4). Here, (i,j) denotes that the nodes indexed *i* and *j* are selfish in this run of simulation. The two separate simulations are executed 50 times with different random seeds. The results of the simulations are depicted in Figure 9 and Figure 10.

As shown in Figure 9 and Figure 10, all three metrics in two sets of simulations fluctuate to some extent, which indicates the importance of selfish node combinations in MANETs. It reflects that even with the same proportion of selfish nodes in the network, the impact of selfish nodes is significantly different.

**Figure 11:**
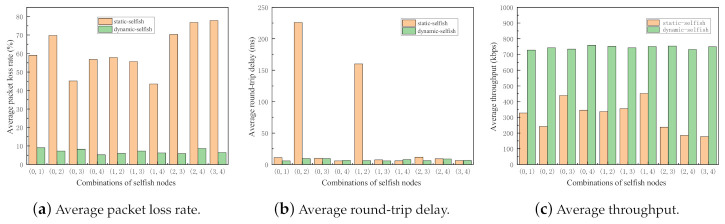
Performance impact of different combinations of selfish nodes following the same mobility model (5 nodes). Every node has the same mobility in each run, but there are different selfish node combinations.

In order to investigate the impact of selfish node combinations with the same node mobility, the following simulation is carried out. In the simulation, the same random seed is used for each combination of selfish nodes, which ensures the randomness is exactly the same in each simulation. The main purpose of this simulation is to investigate the differences among different selfish node combinations under the same node mobility in terms of the three evaluation metrics. The results of the simulation are illustrated in Figure 11.

From the simulation results shown in Figure 11, we can clearly observe that the average packet loss rates of the network with static selfish nodes significantly varies with different combinations of selfish nodes. It decreases to the lowest value, 43.5484%, when node(1) and node(4) are selfish. However, it reaches the highest value, 77.8226%, when node(3) and node(4) are selfish. Even worse, the average round-trip delay of the network with static selfish nodes is affected seriously by the combinations of selfish nodes. Its highest value is 225.86ms while the lowest value is 5.80994 ms in the simulation. The other evaluation metrics of both networks with dynamic and static selfish nodes are also influenced by the combinations of selfish nodes, although the impact is not that serious.

The different of selfish node combinations cause significant performance decrements, which implies that some nodes play more important roles in MANETs than others because of the different positions, movement, and speeds. The performance of the network critically reduces when the key nodes are selfish in MANETs, as shown Figure 1 in Section 1.

## 7. Discussion

The objective of this study was to quantitatively analyze the impact of energy consumption based dynamic selfish nodes, comparing with that of the static and altruistic ones, in MANETs. The computer simulation based experimental results in this work demonstrate that the impact of the selfish nodes is strictly influenced by their mobilities, densities, proportions, and combinations in terms of average packet loss rates, round-trip delay, and throughput. The results suggest that node mobility models and the proportion of selfish nodes are the most influential factors that impact selfish nodes. The node densities and selfish node combinations are not negligible factors when investigating node selfishness in the network. Especially, in some subclasses of MANETs, e.g., VANETs, where nodes behave in a more organized fashion, these two factors should not be underrated. Generally, the more selfish nodes involved in the network, the worse the network performance is in MANETs. Nevertheless, in a dense network, the communication performance is slightly worse because of the link breakages caused by the high node mobility. These results should be taken into account when considering how to detect or incentivize selfish nodes in MANETs. Furthermore, the dynamic selfish nodes are less harmful to the performance of the network, but they are more realistic than the static ones.

The reliability of these data is partially limited by the simple analog model type, unit-disk model. In theory, more complex analog models are more accurate but computationally intensive. The unit-disk analog model is suitable for wireless simulations in which the details of the physical layer are not important. Therefore, the data produced by the simulation based on the unit-disk model are plausible and adequate to this work at the present stage.

There are some threats to the validity of our experimental results. Internally, the parameter setting in our experiments is an influential factor of the validity of results. Especially, the energy consumption parameters could affect the number of dynamic selfish nodes in the network. Externally, the physical environment (e.g., buildings, mountains, etc.) around mobile nodes in MANETs has a great impact on the link quality of wireless communications. It becomes more realistic that environmental obstacles are considered in the simulations. Intrinsically, the randomness produced by the finite number of random seeds is hard to cover all of probabilities. In our experiments, each simulation was executed 50 times with different random seeds, and the average values are plotted in the figures. It ensures the validity of the results as much as possible.

Further studies should take into account the integrated consideration of mobility, density, proportion, and combination of selfish nodes with different weights in MANETs. It is a solid base for the detection and incentive mechanisms of selfish nodes, which can improve network performance by fully utilizing all possible resources in the networks.

## 8. Conclusions and Future Work

The selfishness caused by limited energy brings harmful impacts on the network performance of MANETs, which highly depend on cooperative communications. Understanding the impacts of the selfish nodes in MANETs paves the road to the selfish nodes detection and incentive mechanisms that could improve the overall network performance.

In this study, the impacts of energy consumption based dynamic selfish nodes in MANETs are quantitatively analyzed based on extensive computer simulations. The results of our experiments suggest that there are significant impacts of selfish nodes in MANETs in terms of packet loss rate, round-trip delay, and throughput, no matter what kind of selfishness. Our quantitative analysis concluded that selfish node proportion and mobility have more significant impacts on the network performance in MANETs in terms of the metrics. The impact of dynamic selfish node densities is not as serious as static ones. The selfish node combinations in MANETs indirectly reflect the impacts of the mobility model and selfish node density. In general, the more selfish nodes involved in the network, the worse network performance is in MANETs. However, in an extremely dense network, the routing protocol deals with much more discovery and maintenance tasks, which lead to longer round-trip delays between the sender and the receiver. Static selfish nodes bring more harmful effects to the network than dynamic ones because more packets are dropped by the static selfish nodes. However, dynamic selfishness based on energy consumption is more close to the reality than static selfishness. It is crucial to accurately evaluate the impact of node selfishness so that it provides a base to develop effective detection and incentive mechanisms.

By all counts, and with proven results, it is no wonder that the four characteristics, namely mobility, density, proportion, and combination, of selfish nodes affect the network performance to different degrees. The joint consideration of these characteristics of selfish nodes in MANETs will open more interesting research topics, e.g., wireless routing algorithms, mobile nodes cluster head selections, mobile edge computing-based data offloading, and so forth.

Based on this work, to design a more accurate and fairer selfish node detection mechanism in MANETs is our nearest research plan. Furthermore, a more effective selfish node incentive mechanism that could fully utilize the network resources in MANETs is our final research goal.

## Figures and Tables

**Table 1:** Network parameters.

Parameters	Values
Simulation Time	500 s
Simulation Area	300 m × 1000 m
Number of Nodes	up to 50
Transmission Range	250 m
Mobility	Stationary, Random Waypoint
Node Speed	20∼50 m/s
Packet Size	100 bytes
Packet Rate	1 Pkt/s
Routing Protocol	AODV
Bit Rate	2 Mbps
NIC	*IdealWirelessNic*

**Table 2:** Energy consumption parameters. (P.C. stands for power consumption).

Parameters	Values
Nominal Capacity	0.05 J
Shutdown Capacity	0 J
Start Capacity	0.025 J
Initial Capacity	0 J ∼ 0.05 J
Power Generation	1 mW
Off P.C.	0 mW
Sleep P.C.	1 mW
Switching P.C.	1 mW
Receiver Idle P.C.	2 mW
Receiver Busy P.C.	5 mW
Receiver Receiving P.C.	10 mW
Transmitter Idle P.C.	2 mW
Transmitter Transmitting P.C.	100 mW
